# 

**Published:** 1888-03

**Authors:** 


					﻿1888.
OLD SERIES
Vol. xxv
NEW SERIES J
Vol. V.-No. I.

JI® (JOURNAfcsfe/^



W.F.WESTMORELAND.M Dj
H.V.M.MILLER.M.D.LL.D.
Published ky james p.harrison&ck
kffi.____________*flTLANTA,GA. . A4
SUBSCRIPTION: S2.5O A YEAR.
				

